# Notes and News

**Published:** 1896-11-21

**Authors:** 


### NOTES AND NEWS.

(Collected and Communicated.)

We would remind our readers that the voting papers for the election of members of the General Medical Council will be distributed on or about November 25th, and that the last day for their return will be December 5th. The poll will be declared on December 12th.

The new Verneuil Home for Consumptives at Verneuil, France, is now open. It is splendidly situated amidst pine woods, and faces the sea, at between Pouliguen and La Baule in Brittany. Two hundred guests attended the inaugural banquet.

Montreal will be quite flooded with science in August and September next year, for then the British Association meeting terminates on the 26th of the first-named month, and that of the British Medical Association commences on the 31st.

Diphtheria still prevails at Lewisham. Stringent measures are being taken by the sanitary authority, but without marked results at the moment, though the number of deaths within the last few days has been small. The Board Schools have not been closed but the attendance has been very small.

We understand an action is pending between the Great Northern Central and King's College Hospitals in respect to a legacy of £500 left in his will by the late Sir Francis Lycett to "King's Cross Hospital." There being no hospital of this title the King's College authorities claim that their institution was meant, "Cross" having been written in mistake for "College"; while the Great Northern Central officials base their claim on the fact that formerly their institution went by the name of the "King's Cross Hospital," under which title it was known by Sir Francis Lycett. By the time the legal authorities have come to a decision on the matter it does not seem likely that there will be much of the legacy left for either party.

The hospitals are now benefiting through the liberality of the Prince of Wales, who is sending his annual presents of game to the various institutions.

Dr. Koch and Dr. Kohlstock are on their way to South Africa to investigate, at the request of the Cape Government, the outbreak of rinderpest. They will establish their laboratory at Cape Town.

Guy's Hospital has just sold a piece of their property in Nelson Street, Bermondsey, for the sum of £4,600 to the London County Council to be laid out as an open space for the good of the population.

The Chelsea Guardians appear to be very active in building operations. The Nurses' Home in connection with the Chelsea Infirmary has just been completed, and now we learn that they are about to erect a new workhouse in Arthur Street. The same firm of architects will have charge of the work.

The new extension of the Jews' Hospital and Orphan Asylum, West Norwood, is to be opened in the spring by the Duke of Cambridge. It is stated that the Lord Mayor and Sheriffs will attend in state on the occasion, and that a dinner will be held at the Mansion House afterwards.

A department for treatment by electricity has been started at the Royal Edinburgh Infirmary. Dr. Milne Murray, who already holds a post at the infirmary, has undertaken the superintendence of the department, with Dr. Dawson Turner as his honorary assistant.

The deadlock at Manchester in relation to the David Lewis Trust and the two hospitals concerned does not appear likely to be removed immediately. At the same time it is creating an immense amount of interest, and it is to be hoped that concessions on the part of the Trust Committee and that of St. Mary's Hospital will be made, and the difficulties solved.

Mr. W. T. Smedley, secretary of the Birmingham Hospital Saturday Fund, has been presented with an illuminated address and album containing portraits of the members, by the Executive Committee of the fund, in recognition and appreciation of his untiring labours on behalf of the movement, and as a mark of the personal esteem in which he is held.

An extraordinary incident has recently occurred in connection with the Queen's Hospital, Birmingham. Through an accident, the body of one deceased person was interred in mistake for that of another. The mistake arose through the officiousness of an undertaker who went to the mortuary of the hospital without waiting to be accompanied by the porter, and took away the only body he saw, which was accordingly buried under a wrong name. The mistake was found out in the evening of the same day, but owing to a difficulty in finding the address of the undertaker who had made the mistake, the wrong body was interred. Meanwhile, the body that should have been interred remained at the mortuary. The widow of the man Kemp, who was wrongly interred, has signed a petition to the Home Secretary, in which she desires that her husband's body may be disinterred.

